# The Effectiveness of transperineal template guided mapping biopsy compared to transrectal ultrasound guided biopsy in detecting prostate cancer

**DOI:** 10.1186/1753-6561-9-S1-A13

**Published:** 2015-01-14

**Authors:** RS Dunne, DM Bouchier-Hayes

**Affiliations:** 1 Royal College of Surgeons in Ireland, 123 St. Stephen’s Green, Dublin, Ireland; 2 Galway Clinic, Doughiska, Galway, Ireland

## Background

To assess the effectiveness of transperineal template guided mapping biopsy (TTMB) in detecting prostate cancer in patients with previous multiple negative transrectal ultrasound guided (TRUS) biopsies of the prostate.

## Methods

From April 2011 to February 2013 22 patients who had previously undergone 2 or more TRUS biopsy with a continuing rising PSA were biopsied using an anatomically guided template. Clinical parameters included age, initial PSA, PSA pre TTMB biopsy and number of previous biopsies. Number of cores sampled, number of cores positive for cancer and Gleason score were assessed. Results: Average age is 61.45 years with average of 2.5 previous biopsies and pre biopsy PSA of 15.5. On average 22.3 cores were sampled in each patient. PSA rose 6.4 ng/ml between initial biopsy and PSA triggering TTMB. Average elapsed time from initial biopsy to TTMB was 3.3 years. Overall 31.8% of patients (n=7) were diagnosed with prostate cancer at TTMB. There were 4 patients found to have Gleason 3+3=6, 1 Gleason 3+4=7 and 4 Gleason 4+4=8.

## Conclusions

TRUS biopsy is known to miss clinically significant pathology of the prostate. In this single surgeon single institution analysis we examined the efficacy of the TTMB in identifying prostate cancer in patients with multiple previous negative biopsies and a rising PSA, and found over 30% of patients with previous multiple negative biopsies to have clinically significant prostate cancer. This study demonstrates that in the right hands TTMB can be an integral step in the management pathway of patients with suspected prostate cancer.

